# Diffusible GRAPHIC to visualize morphology of cells after specific cell–cell contact

**DOI:** 10.1038/s41598-020-71474-0

**Published:** 2020-09-02

**Authors:** Nagatoki Kinoshita, Arthur J. Y. Huang, Thomas J. McHugh, Atsushi Miyawaki, Tomomi Shimogori

**Affiliations:** 1 Molecular Mechanisms of Brain Development, Center for Brain Science (CBS), RIKEN, Saitama, Japan; 2 Exploratory Research for Advanced Technology (ERATO), Japan Science and Technology Agency (JST), Tokyo, Japan; 3 Circuit and Behavioral Physiology, CBS, RIKEN, Saitama, Japan; 4 Cell Function Dynamics, CBS, RIKEN, Saitama, Japan

**Keywords:** Biological techniques, Neuroscience

## Abstract

The ability to identify specific cell–cell contact in the highly heterogeneous mammalian body is crucial to revealing precise control of the body plan and correct function. To visualize local connections, we previously developed a genetically encoded fluorescent indicator, GRAPHIC, which labels cell–cell contacts by restricting the reconstituted green fluorescent protein (GFP) signal to the contact site. Here, we modify GRAPHIC to give the reconstituted GFP motility within the membrane, to detect cells that make contact with other specific cells. Removal of leucine zipper domains, located between the split GFP fragment and glycophosphatidylinositol anchor domain, allowed GFP reconstituted at the contact site to diffuse throughout the entire plasma membrane, revealing cell morphology. Further, depending on the structural spacers employed, the reconstituted GFP could be selectively targeted to N terminal (NT)- or C terminal (CT)-probe-expressing cells. Using these novel constructs, we demonstrated that we can specifically label NT-probe-expressing cells that made contact with CT-probe-expressing cells in an epithelial cell culture and in *Xenopus* 8-cell-stage blastomeres. Moreover, we showed that diffusible GRAPHIC (*d*GRAPHIC) can be used in neuronal circuits to trace neurons that make contact to reveal a connection map. Finally, application in the developing brain demonstrated that the *d*GRAPHIC signal remained on neurons that had transient contacts during circuit development to reveal the contact history. Altogether, *d*GRAPHIC is a unique probe that can visualize cells that made specific cell–cell contact.

## Introduction

One of the ultimate goals of cell biology is to clarify the cell–cell contact of individual cells and to understand how these contacts influence their final position and function. Especially in neuroscience, clarifying the connectivity of individual neurons helps us understand how these circuits process information, drive perception, and control behavior. In the past 50 years, many anterograde and retrograde tracers have been developed to reveal neural fiber trajectories and their target neurons ^1,2^. These methods have brought enormous insights regarding neural projections and now the connectivity of the majority of large fasciculated fibers has been mapped. An ideal tracer for visualizing neuronal connectivity is trans-synaptic, but it is limited to direct contact and regulated via genetic techniques. Wheat germ agglutinin and tetanus toxin C fragment meet these criteria^3–5^, however, the limitation of label extension via synaptic connections can lack the resolution needed to identify individual neuronal connectivity. Herpes simplex virus-1 and 2^6^ and pseudorabies virus^7^ also spread trans-synaptically and polysynaptically. The modified rabies virus systems label has only one-step trans-synaptic connections ^8–10^, however, it suffers from the general problems of virus-based systems, such as cytotoxicity (especially with long-term observation), differences in infection efficiency among cell types, and preference in labeling direction (anterograde or retrograde).

Recently, various protein probe systems have been reported to visualize synaptic connections via trans-synaptic molecular interactions between pairs of pre- and postsynaptic membrane proteins. ID-PRIME (Interaction-Dependent PRobe Incorporation Mediated by Enzyme) labels synapses by trans-synaptic enzyme–substrate reactions^11,12^. As such, this method requires delivery of chemical compounds to synaptic sites. GRASP (Green fluorescent protein [GFP] Reconstitution Across Synaptic Partners) ^13^ and its mammalian-optimized version, mGRASP ^14^, is generated based on the BiFC (Bimolecular Fluorescence Complementation) technique ^15,16^. Here, split GFP fragments tethered to pre- and postsynaptic membrane proteins reconstitute a GFP molecule in the synaptic cleft when a synapse is formed. Among these methods, we also have generated novel indicators for intercellular contact that we have termed GRAPHIC (Glycophosphatidylinositol [GPI] anchored Reconstitution-Activated Proteins Highlight Intercellular Connections) ^17^. GRAPHIC shows intercellular connectivity by GFP reconstitution, but its molecular structures are quite different from GRASP. This allows the GRAPHIC series to produce many variations in the connectivity signal pattern in different systems, not only in neuroscience. Although these methods are powerful tools for identifying precise locations of synaptic connections, they are associated with some difficulties in identification of connected neurons to trace their trajectories and morphologies.

In this paper, we sought to modify GRAPHIC to make reconstituted GFP molecules membrane-diffusible and determine whether this would allow us to image the morphology of the cell body based on cell–cell contact. To make the GRAPHIC membrane diffusible, we removed the leucine zipper domains that are located between the split GFP fragments and the GPI anchor domain to facilitate and stabilize GFP reconstitution. Removing the leucine zipper domain resulted in the reconstituted GFP signal spreading out within the plasma membrane and delineating the entire cell morphology. We termed this system *diffusible* GRAPHIC (*d*GRAPHIC) to distinguish it from the original GRAPHIC. The splitGFP technique is based on the auto-assembly of two GFP fragments split with in 11 ß-sheets (GFP1-10 and GFP11, GFP1-7 and GFP8-11, GFP1-8 and GFP9-11), non-fluorescing on their own but self-reconstitution form a fluorescent molecule. The fundamental probe design for the *d*GRAPHIC contains NT-probe (split sites with the 1–7 N terminal) and CT-probe (split sites with the 8–11 C terminal) (CT) probe fragments, followed by a Thy-1 GPI anchor domain.

Interestingly, in the *d*GRAPHIC system, the reconstituted GFP showed a distribution preference for NT-probe-expressing cells. However, adding spacer domains to CT probes reversed the unidirectional distribution of the reconstituted GFP onto the plasma membrane of CT cells. First, we applied GRAPHIC to *Xenopus* 8-cell-stage blastomeres (the NT probe is injected into the right animal-smaller blastomere, and the CT-probe is injected into the left animal-smaller blastomere) and showed that reconstituted GFP was distributed only on NT-probe-injected cells. We also asked whether GFP reconstitution occurs only with a small contact site and can distribute over the entire cell membrane to delineate cell morphology. To test this idea, we used a nervous system that makes cell–cell contact only at small synaptic sites. We showed that GFP reconstitution occurs at the contact site and provides a sufficient GFP signal to trace back entire neurons using the in vivo thalamus-cortex system. These results indicate that *d*GRAPHIC can be used to label cell–cell contact and isolate cells that made contact in many different systems.

## Results

### *d*GRAPHIC design and spatiotemporal dynamics

**Figure 1:**
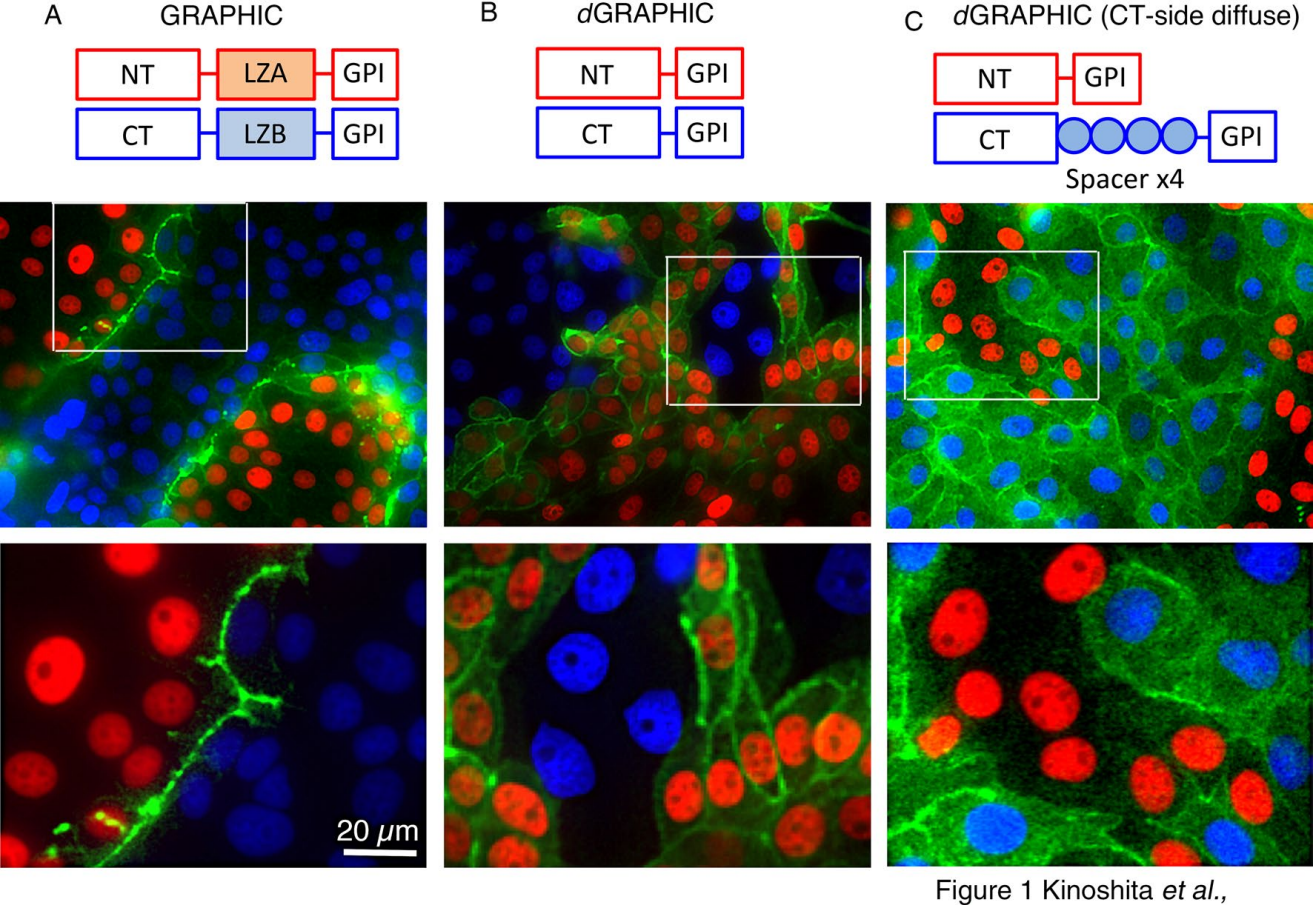
Distribution of reconstituted GFP is dependent on GRAPHIC probe molecular structure. A red nucleus (labeled by H2B-mCherry) indicates an NT cell, and a blue nucleus (labeled by H2B-Azurite) indicates a CT cell. (**A**) GRAPHIC: NT probe containing an acidic leucine zipper domain (LZA) and CT probe containing a basic leucine zipper domain (LZB). Reconstituted GFP was observed only at the boundary between NT and CT cells. White box indicates position of higher magnification view. (**B**) *d*GRAPHIC: Leucine zipper domain was removed from both NT and CT probes. *d*GRAPHIC showed almost all reconstituted GFP signals on NT cells, compared with CT cells. (**C**) A probe pair consisting of NT and CT probes with 3 spacer domains (15 aa × 3) inserted between the GFP-CT fragment and GPI anchor domain. Reconstituted GFP was equally observed on both NT and CT cells. (**D**) Combination of NT and CT probes with 4 spacer domains (15 aa × 4). Reconstituted GFP was detected predominantly on CT cells. Scale bar, 40 µm and for higher magnification 20 μm.

GRAPHIC is a set of two non-fluorescent GFP fragments displayed on the plasma membrane, which reconstitute as a fluorescent GFP molecule when two cells presenting each fragment make contact with each other ^17^. The fundamental probe design contains a mouse NT preproacrosin 24 aa leader sequence followed by a split-GFP fragment, an acidic leucine zipper domain (LZA) for the NT probe, basic leucine zipper domain (LZB) for the CT probe, and mouse Thy-1 GPI anchor domain (CT 31 aa) (Fig. 1A). GFP contains 11 ß-sheets with split sites for GRAPHIC being located between the 1–7 (NT probe) and 8–11 (CT probe) fragments (1–157 and 158–238 amino acid residues of sfGFP, respectively). For identification of NT cells, nuclei-localized mCherry protein (human histone (H2B)-mCherry) was fused to the NTprobe with the self-cleavable T2A peptide, for CT cells, nuclei-localized Azurite protein was fused to the CT probe with T2A (Fig. 1). As shown previously, the reconstituted GFP signal is located specifically at the contact site of each cell and no diffusion of GFP was observed by GRAPHIC (Fig. 1A).

When we generated GRAPHIC in our previous work, the idea of putting a leucine zipper domain in both NT and CT probes was to facilitate GFP reconstitution because no endogenous receptor-ligand molecular interactions exist in the GRAPHIC system. This worked well and produced an intense GFP signal at the contact site ^17^. Because the fluorescence intensity obtained from reconstituted GFP in the GRAPHIC system was unexpectedly strong, it made us think that it would still provide a strong GFP signal even if GFP is spread out and not localized at the contact site. A leucine zipper domain was inserted to facilitate and stabilize protein–protein interaction in GRAPHIC, therefore to test this idea, we removed the leucine zipper domain from both probes to test whether the modification increased diffusion in epithelial cells (LLCPK1). Reconstituted GFP was spread out over the plasma membrane and delineated cell morphology. Unexpectedly, the GFP signal was specifically observed on NT-cell surfaces but not on CT cells (Fig. 1B).

To reveal the basic mechanism of *d*GRAPHIC that controls the unidirectional distribution of reconstituted GFP, we introduced spacer domains between the CT fragment and GPI anchor domain (Fig. 1C). Addition of multiple glycine-rich spacers (GGGGSGGGGSGGGGS x n) is considered to lengthen the distance between membranes and GFP fragments and allow GFP fragments to move flexibly. Insertion of tandem spacers into the CT-probe resulted in a detectable signal on both CT and NT cells, suggesting that the reconstituted GFP distribution became bidirectional (data not shown). However, this signal intensity was substantially lower (data with the same signal intensity are not shown) than that of the non-spacer-containing CT-probe set (Fig. 1B), which may be caused by signal dilution via expansion of the distribution area. Insertion of four spacers into the CT probe dramatically reversed the unidirectional distribution of reconstituted GFP between NT cells and CT cells (Fig. 1C). These results indicate that reconstituted GFP can diffuse bilaterally depending on the CT-probe spacer length. We also tested whether insertion of spacer domains in NT probes changes the GFP distribution, however, it did not (data not shown). To understand how GFP can diffuse on the membrane, we changed the GPI anchor domain to the trans-membrane domain of platelet-derived growth factor receptor (PDGFR-TM). Reconstituted GFP is preferentially transferred to CT cells by using PDGFR-TM pairs (Supplementary Fig. S1). However, all chimera combinations of PDGFR-TM type and GPI anchor type probes showed a stronger reconstituted signal on cells expressing the GPI anchor-type probe (Supplementary Fig. S1). We tested more than 150 combinations of probe pairs containing PDGFR-TM molecules, and all of them showed some signal diffusion. Therefore, we think that it may be a general feature that the intercellular membrane protein complex transfers to either contacted cell membrane, however, the directional preference depends on its molecular structure. Although, we do not have clear idea for the mechanism and theoretical backbone for how *d*GRAPHIC diffuses, we still thought *d*GRAPHIC will be a useful unique probe to detect cell–cell contact in many different biological contexts.

**Figure 2:**
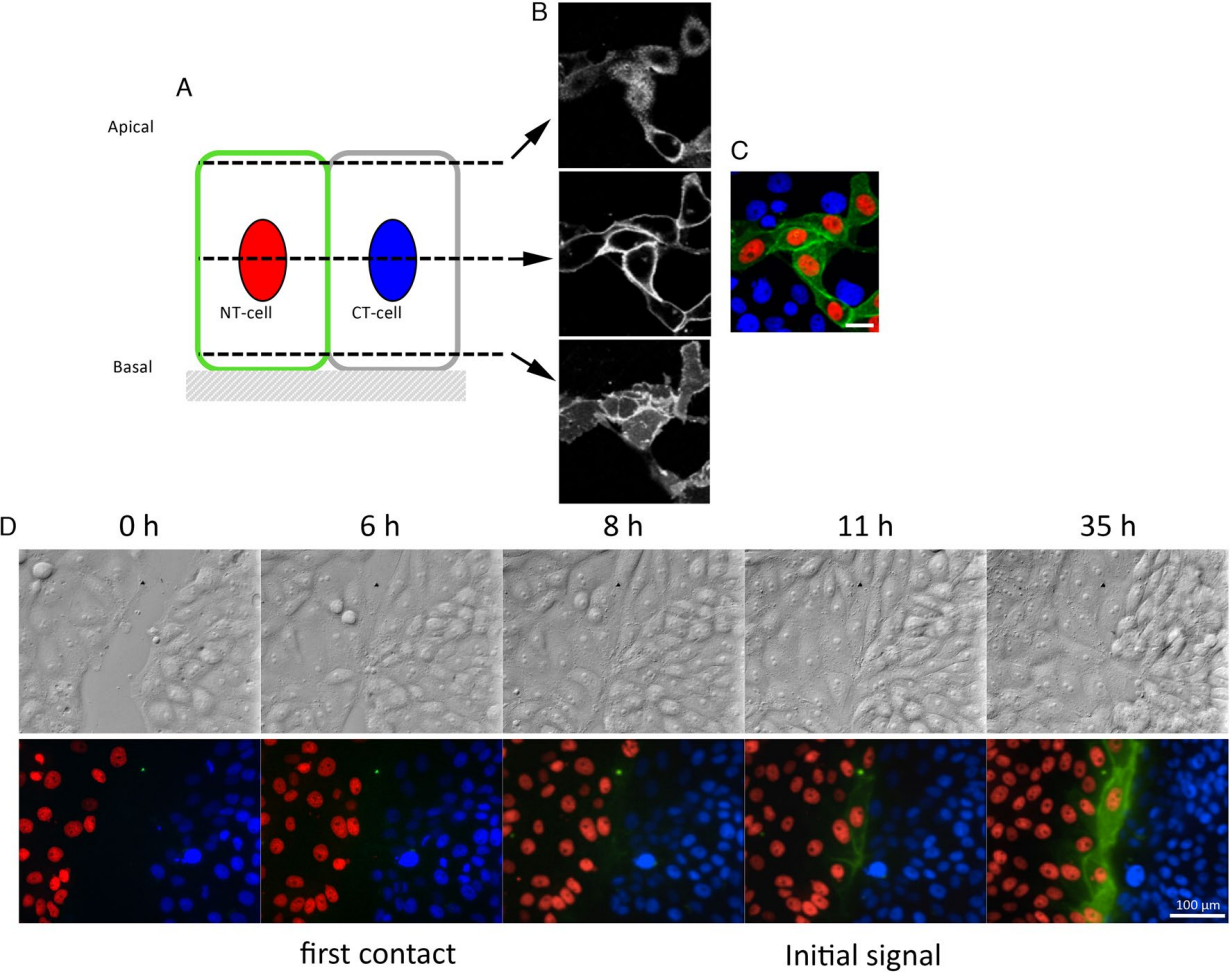
Spatiotemporal characteristics of *d*GRAPHIC. (**A**) Confocal images reveal subcellular distribution of reconstituted GFP in co-cultured NT-probe-expressing and CT-probe-expressing LLCPK1 cells. Schema of z-positions of confocal images. (**B**) Stacked confocal images (z = 1.2 μm) at apical surface (top), middle position (center), and basal membrane (bottom). (**C**) Full stacked image (z = 7.2 μm) of reconstituted GFP, nuclei of NT-cells (red), and nuclei of CT-cells (blue). Scale bar, 20 µm. (D) Time lapse images for the generation of reconstituted GFP signal between NT-LLCPK1 cells (red nuclei) and CT-LLCPK1 cells (blue nuclei). Upper panels are bright field (differential interference contrast) images of the bottom fluorescence images. The reconstituted GFP signal was generated at cell–cell contact sites and gradually spread out over the whole plasma membrane of contacted NT cells (red nuclei). Scale bar, 100 μm.

To further characterize the nature of *d*GRAPHIC, we analyzed the subcellular distribution of reconstituted GFP. Confocal images were taken and different z-positions in a co-culture of NT-probe- and CT-probe-expressing LLCPK1 cells were observed (Fig. 2A–C). Cross-sectional images clearly demonstrated that reconstituted GFP was displayed on the plasma membrane and not within the cytoplasm (Fig. 2B). Next, to determine the earliest time point for detection of *d*GRAPHIC signals after cell–cell contact, we performed time-lapse imaging using the LLCPK1 cell line. The *d*GRAPHIC signal was first detected 5 h after initial contact, and its intensity gradually increased within the membrane of contacted cells over the next 24 h (Fig. 2D and Supplementary movie 1). Moreover, in this movie, it is clearly shown that GFP reconstitution starts at the contact site (around 7 s into the movie), and GFP starts to spread to the cell membrane (8–10 s), however, there is no GFP diffusion happening to cells located away from the contact site. There is a cell that proliferated from a GFP positive cell close to the contact site, and moved away from the contact site (11 s). From this movie, it is suggested that the reconstituted GFP is restricted to NT cells that had been in contact, and migration of GFP positive cells or proliferated cells from GFP positive cells cause GFP distribution to spread from the contact site. Next, to determine the *d*GRAPHIC signal stability, we also observed GRAPHIC signal dynamics when intercellular contacts were disrupted by ion chelation (data not shown). Similar to our previous results, reconstituted GFP was not abolished, but rather stably remained on the NT-probe-expressing cells ^17^.

### Application of *d*GRAPHIC to visualize contacted cells

**Figure 3:**
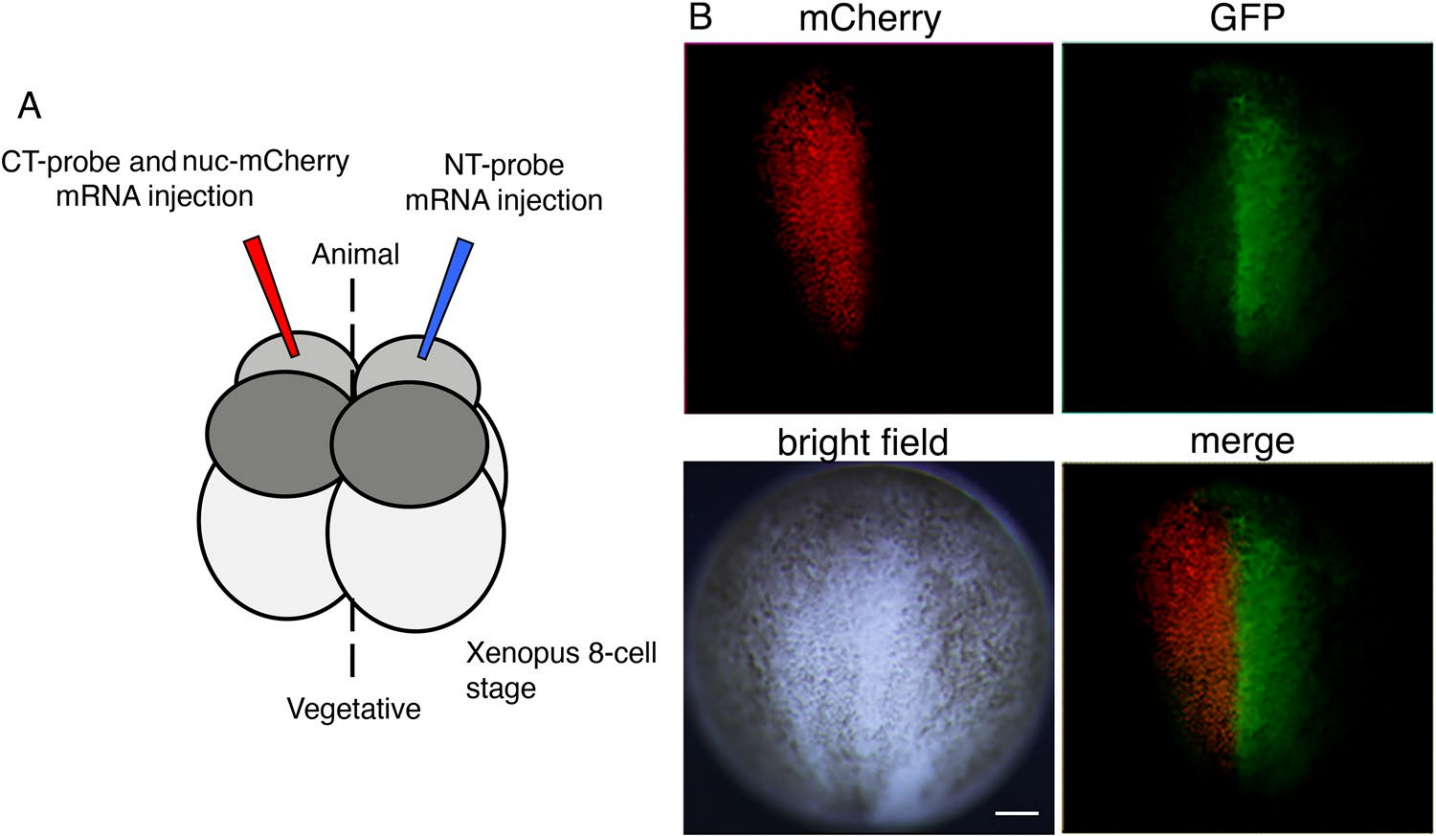
*d*GRAPHIC application in *Xenopus* blastomere. (**A**) Schema of mRNA injection into 8-cell-stage *Xenopus* blastomeres. The mRNA of the NT probe is injected in the right animal-smaller blastomere of the *Xenopus* 8-cell stage, and the mRNAs of the CT probe and nuc-mCherry are injected together into the left animal-smaller blastomere. (**B**) Embryos are grown to the early neurula stage. Cells expressing the CT probe (red nuclei) are present only within the left half of the neural plate, while GFP signals can be detected only in the right half of the neural plate. Scale bar, 100 μm.

To test whether *d*GRAPHIC can detect cell–cell contact in vivo, we first used a *Xenopus* oocyte, which allowed us to microinject mRNA into individual blastomeres that will give rise to different organs and tissues ^18^. The mRNA of the NT probe is injected in the right animal-smaller blastomere of the *Xenopus* 8-cell stage, and the mRNAs of CT probe and nuc-mCherry are injected together into the left animal-smaller blastomere (Fig. 3A). Reconstituted GFP signals can be detected at an early neurula stage (Fig. 3B). Cells with red nuclei (expressing the CT probe) are restricted to the left half of the neural plate, while GFP signals can be detected only in the right half of the neural plate.

Although strong signals are observed near the midline where each probe-expressing cell made contact, there are also GFP-positive cells distributed away from the midline. From Supplemental Movie 1, it is suggested that this GFP spread is from contacted GFP positive cell movement or proliferated from GFP positive cells (Supplementary Movie 1). When the mRNA of the NT probe is injected in the right animal-smaller blastomere of the *Xenopus* 8-cell stage and only the mRNA of nuc-mCherry is injected into the left animal-smaller blastomere, no GFP signal was observed (data not shown). These results suggest that GFP reconstitution occurs at the contact site, and contacted cells can move away from the contact site while GFP remains expressed on the cell surface.

### Application of *d*GRAPHIC in dissociated hippocampal culture and neural network

**Figure 4:**
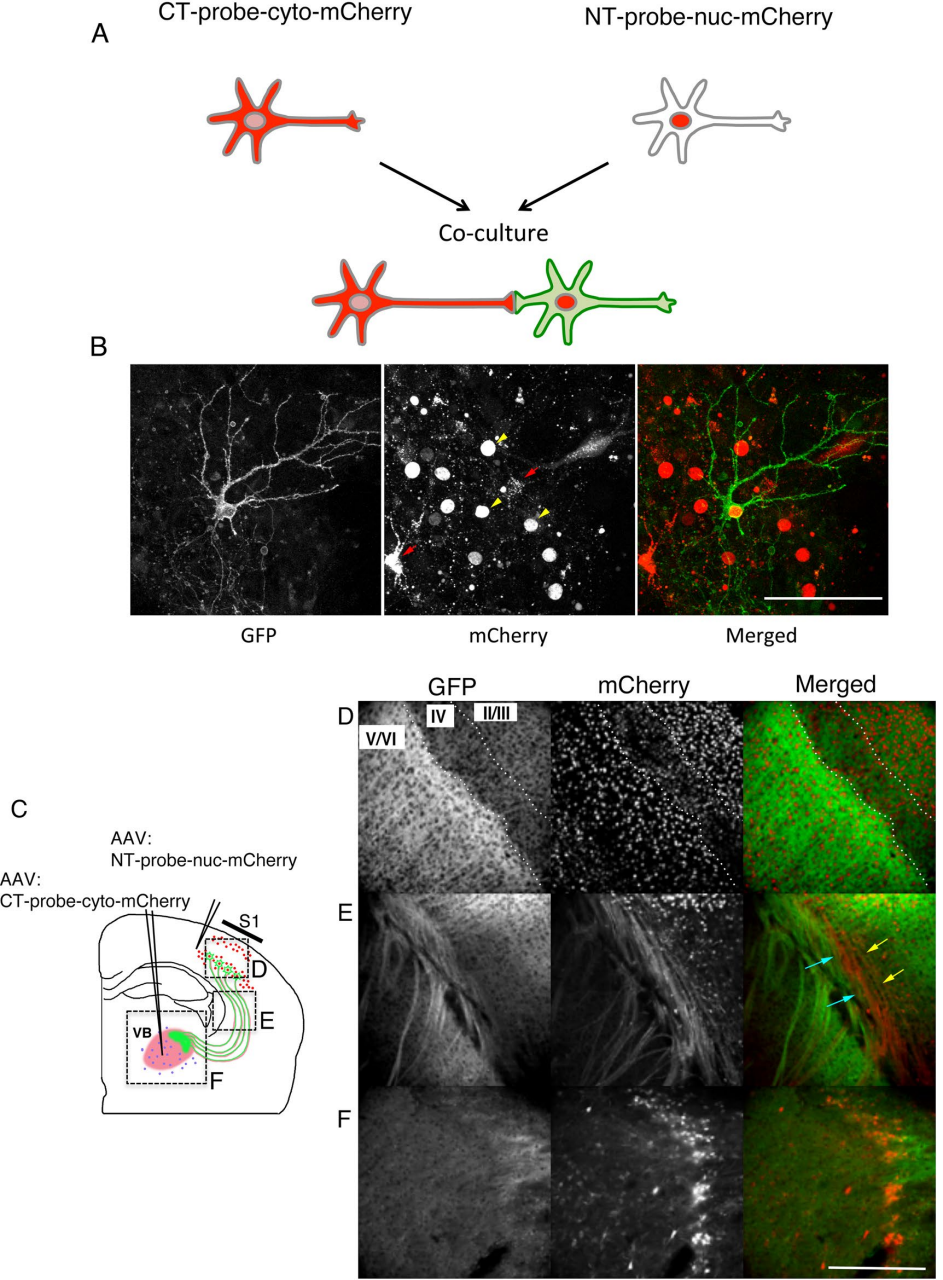
*d*GRAPHIC labels neurons in vitro and in vivo. (**A**) Experimental schema for in vitro *d*GRAPHIC. Each probe plasmid was electroporated into hippocampal neurons in individual cuvettes, and electroporated neurons were mixed and co-cultured. NT neurons show red fluorescence only in the nucleus and CT neurons show red fluorescence throughout the cell. (**B**) Reconstituted GFP in hippocampal cultures. In co-cultured hippocampal neurons, the reconstituted GFP signal was observed only in NT neurons (yellow arrows), which was equally distributed on soma, dendrites, and axons. No GFP signal was present in CT neurons (red arrows). Scale bar, 100 μm. (**C**) Experimental schema for in vivo *d*GRAPHIC. AAVs encoding NT probes (co-expression with red fluorescent nuclear label) and CT probes (co-expression with cytosolic mCherry) were stereotaxically injected into the S1 cortex and VB, respectively. After several weeks, the injected brains were sectioned and observed. (**D**–**F**) In coronal sections, the GFP and mCherry signals were observed in the cortex (**D**), white matter (**E**), and VB thalamus area (**F**). Scale bar, 500 μm.

Our next challenge is to test whether GFP reconstitution occurs at restricted contact sites, such as a synaptic contact site, and whether it still can provide enough GFP to distribute over the entire cell surface to delineate cell morphology. To begin with, we used rat primary dissociated hippocampal neurons ^19^. For identification of NT- and CT-expressing neurons, nuclei-localized mCherry (H2B-mCherry) was fused to the NT probe via a T2A peptide and cytosolic mCherry was coupled to the CT probe with T2A (Fig. 4A). Each probe was electroporated separately into hippocampal neurons in individual cuvettes, and electroporated neurons were mixed and co-cultured (Fig. 4A). Red fluorescence was observed in the nuclei of NT neurons (Fig. 4B, yellow arrowheads), while CT neurons displayed red fluorescence throughout the entire cell (Fig. 4B, red arrows). In co-cultured hippocampal neurons, the reconstituted GFP signal became visible only on NT neurons, equally distributed on the soma, dendrites, and axon (Fig. 4B), demonstrating that *d*GRAPHIC can highlight the whole shape of NT neurons in culture.

To obtain efficiency in a quantitative manner, it is required to reveal specific contact sites, the length of contact, and the area size of the contact site. However, *d*GRAPHIC does not have the ability to measure all of these factors, therefore, it is difficult to obtain the details efficiently. However, one critical factor to reconstitute the detectable amount of GFP is long-lasting contact (at least 5 h, Fig. 2). Second, it does not require a large contact area since reconstitution occurs at a synapse site shown in our previous work^17^. From these, it is likely that, although *d*GRAPHIC has no target sequence for specific contact sites, GFP reconstitution occurs at relatively specific contact sites with relatively stable contact duration rather than short transient contact. Next, we asked whether *d*GRAPHIC can be used in the living brain, and we decided to use the adult mouse cortex-thalamus reciprocal neuronal circuit*,* in which the axons are projected more than 3 mm from the soma. We generated adeno-associated viral vectors (AAV-DJ/8), which separately encode the NT-nuc-mCherry and CT-cyto-mCherry probes and stereotaxically injected them into the cortex (primary somatosensory area, S1) and thalamus (ventrobasal thalamic nuclei, VB) respectively (Fig. 4C). Although AAV-DJ/8 has a strong neuronal transfection ability, it is known to have lower transfection efficiency in mouse cortical-layer IV neurons ^20^, thus, in the S1 area, a strong nuc-mCherry signal was observed in layers V and VI and layers II and III, but only weakly in layer IV (Fig. 4D). NT-probe-expressing neurons in cortical layers V and VI project to the CT-probe-expressing VB thalamus ^21^. Thus, we predicted that GFP is reconstituted within the VB at corticothalamic synapses and is transferred to NT-probe-expressing neurons in layers V and VI. Accordingly, a strong GFP signal was observed throughout cortical layers V and VI (Fig. 4D) and along corticothalamic axons at the entry point to the thalamus (Fig. 4E), while a weaker signal was observed in VB (Fig. 4F). Compared to this result, weaker GFP signals were observed in the upper layers (layers II-IV) which we thought were GFP signals from apical dendrites and branches of Layer V neurons. To confirm this, we used AAV (NT-probe) which has a lower titer rate than AAV-NT-probe-nuc mCherry used in Fig. 4C. We performed the same experiment to describe and observe the GFP positive layer V neurons. In high magnification next to Nissl staining, it is clearly shown there are no GFP positive cell bodies in layer IV and layer II/III but only GFP positive fibers. We never observed GFP positive fibers in the contra-lateral side in these brains, which suggests there are no Layer II/III neurons which have reconstituted GFP (Supplementary Fig. S2).

It is likely that the GFP reconstitution occurred only at the synaptic site between corticothalamic axons and VB neuron dendrites, however, NT-probe-expressing corticothalamic axons (Fig. 4E, blue arrows) and CT-probe-expressing thalamocortical axons (Fig. 4E, yellow arrows) run adjacent to each other and we cannot exclude the possibility that GFP reconstitution occurred at axon-axon interaction sites as well.

### In vivo application of *d*GRAPHIC to visualize individual neuronal morphology

**Figure 5:**
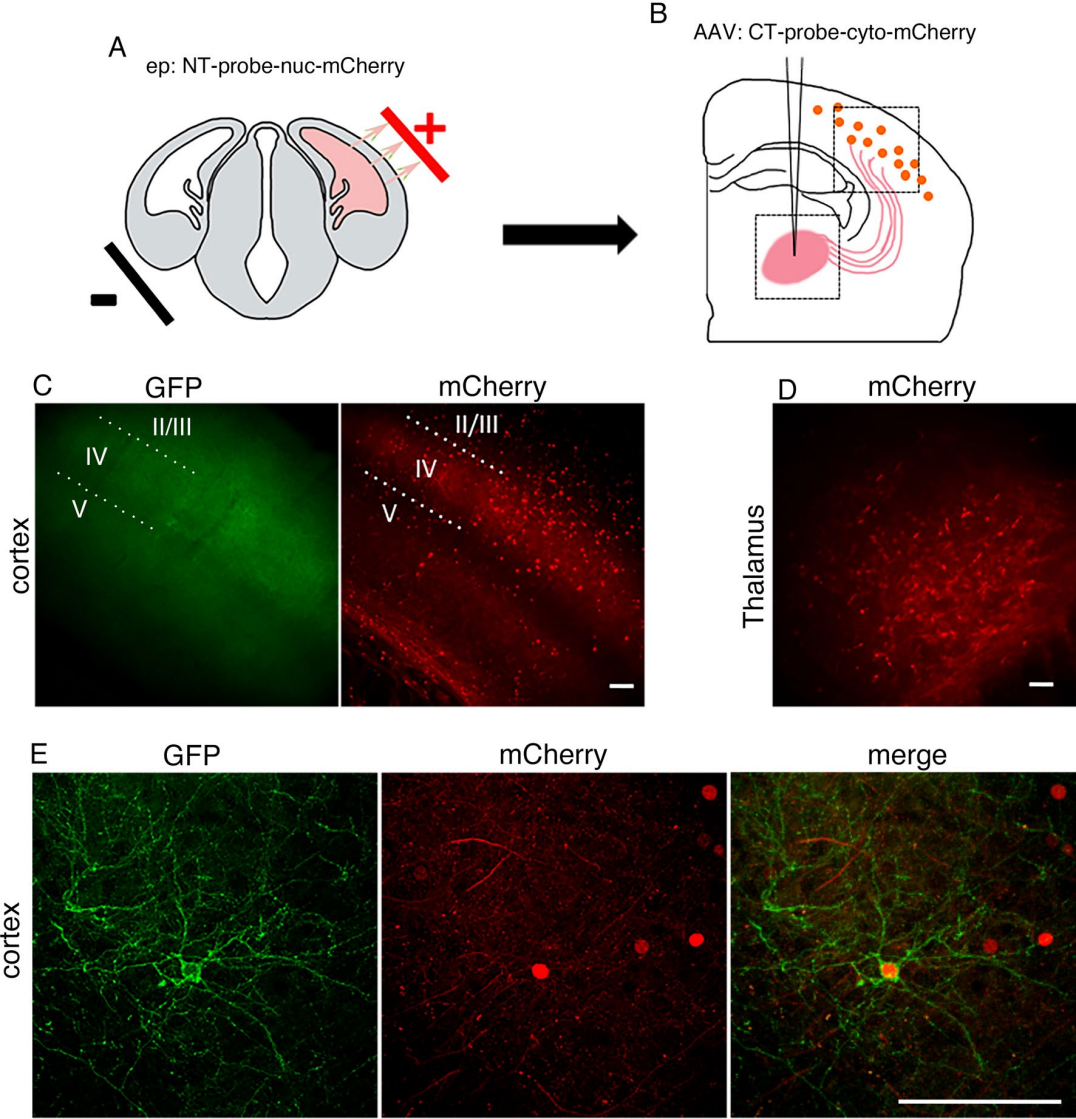
*d*GRAPHIC can reconstitute GFP from synaptic contact. (**A**) In utero electroporation was performed at E13.5 to express NT probes (red nuclear label) in cortical layer IV. (**B**) After the electroporated mice had grown to adult age (about 2 months old), AAV encoding the CT probe (cytosolic mCherry) was stereotaxically injected into the VB. (**C**) Lower magnification images of a sample brain several weeks after injection. Reconstituted GFP and nucleic mCherry signals were observed in layer IV. Scale bar, 50 μm. (**D**) Cytosolic mCherry signal was observed in VB neurons, indicating strong expression of the CT probe. Scale bar, 50 μm. (**E**) Higher-magnification confocal images of layer IV neurons. The reconstituted GFP signal delineates a cortical spiny stellate cell body and dendrites (white arrowheads), which receive input from VB thalamocortical axons. There are also GFP-positive fibers in layer IV, which are processes of other cortical layer IV neurons that received input from VB axons. Scale bar, 100 μm.

To determine whether synaptic contact is sufficient for GFP reconstitution to trace neuron morphology, we next employed in utero electroporation to efficiently and selectively transfect cortical layer IV neurons ^22^. Here, an NT-probe-nuc-mCherry plasmid was electroporated at embryonic (E) day 13.5, targeting cortical layer IV neurons in S1, and embryos were grown to adulthood. As the majority of layer IV neuron dendrites receive synaptic input from VB thalamocortical axons (TCAs) we stereotaxically injected the AAV-CT-cyto-mCherry probe into the VB thalamus of the electroporated animals after postnatal week 6 (Fig. 5A, B). Compared with the dense transfection by AAV injection in layers V and VI (Fig. 5D), in utero electroporation provided sparser NT-probe transfection, primarily in layer IV (Fig. 5C, E). About 1 month after the AAV injection into the VB thalamus, a green fluorescent signal was observed in the cortical layer IV neurons (Fig. 5C and Supplementary Fig. S3) but not in the VB thalamus (data not shown). Reconstituted GFP highlighted the morphology of layer IV neurons with fine processes, characteristic of excitatory cortical neurons that receive thalamocortical inputs in the mouse S1 (Fig. 5E).

Taken together, these data demonstrate that *d*GRAPHIC can provide a GFP signal sufficient to reveal cellular morphology of connected neurons from restricted contact sites in the adult mouse brain. This suggests that the *d*GRAPHIC system is a powerful new tool for dissecting the highly complex connectivity that exists in the mammalian nervous system.

### *d*GRAPHIC to reveal contact history

**Figure 6:**
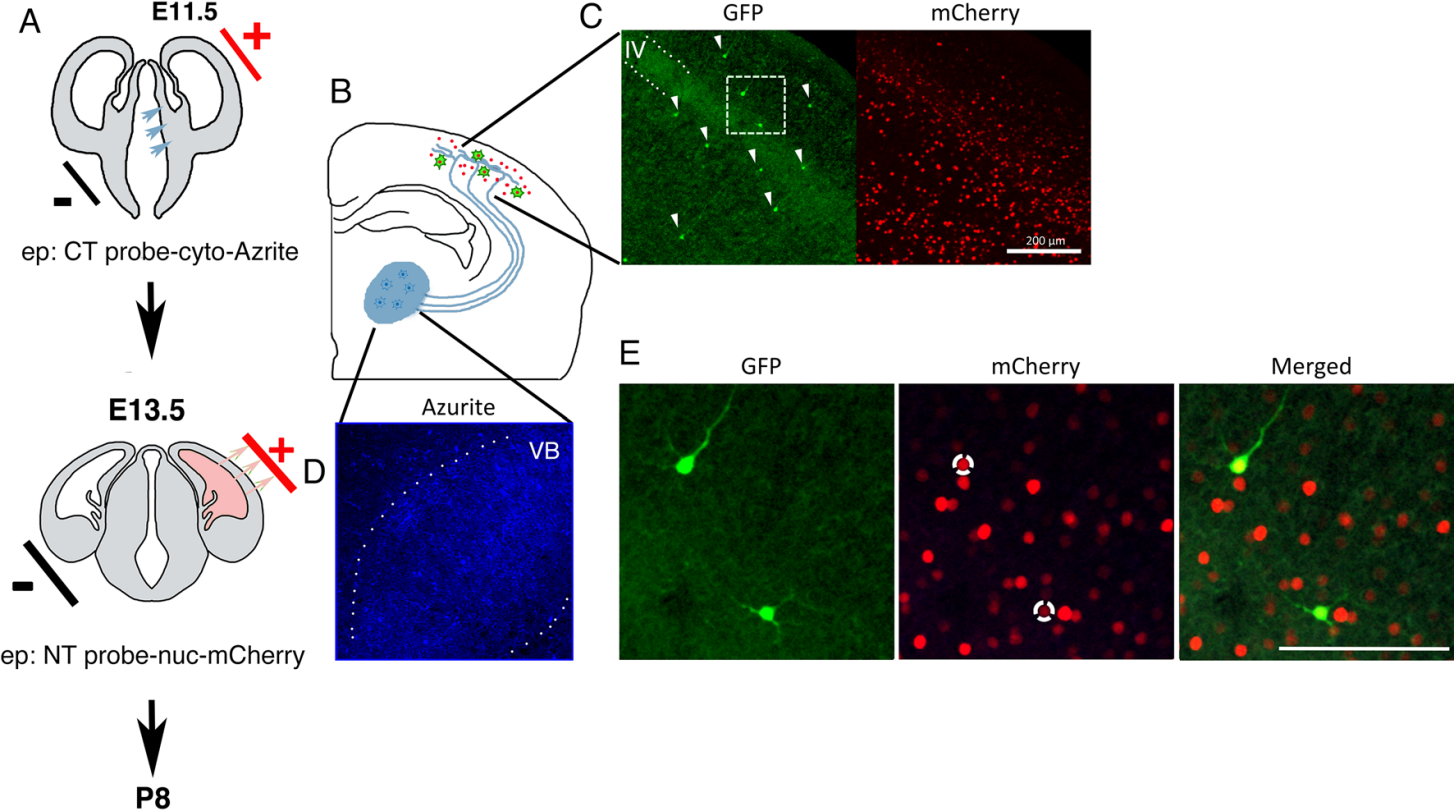
Visualization of past thalamocortical connections in the developing mouse brain. (**A**) Experimental schema. In utero electroporation in the ventricular zone of diencephalon (developing thalamus, blue arrows) was performed at E11.5 to express the CT probe (co-expressed with blue fluorescent nuclear label). Two days later, in utero electroporation in the ventricular zone of telencephalon (pink arrows) was performed at E13.5 to express the NT probe (co-expressed with red nucleus label) in cortical layer IV. (**B**) Schema of thalamocortical axon projections to cortical layer IV in adult brain. (**C**) GFP and mCherry images of the cortical S1 area. Scattered GFP signals are not restricted to layer IV but observed in several cortical layers (white arrowheads). Scale bar, 200 μm. (**D**) CT-probe expression in VB was observed by a blue nuclear signal. (**E**) Higher-magnification images of white dotted box in **C**. Dotted white circles in mCherry image indicate nuclei of GFP-positive neurons. Scale bar, 100 μm.

We have shown that *d*GRAPHIC can be used in mature neuronal circuits to identify neuronal contacts. As these connections are thought to be stable, they provide ample time for reconstitution. However, it remains unknown whether *d*GRAPHIC is suitable for labeling relatively transient connections, such as neuron-neuron contact in an immature neural circuit during development. To test this, we introduced the NT and CT probes into the developing cortex and thalamus, respectively, in the same brain via in utero electroporation. VB thalamic neurons are generated from the ventricular zone of the 3rd ventricle at E11.5, while cortical layer IV neurons are generated in the ventricular zone of the lateral ventricle at E13.5 (Fig. 6A). During development, VB TCAs reach the primary somatosensory cortex around E14.5 and start to innervate the cortex after birth. During the first postnatal days (P0–P1), TCA fibers are diffusely distributed to all cortical layers within S1 and no clustering of axons in layer IV is observed ^23^ (Fig. 6B). In the next few days (P3-P5), TCAs are refined into periodic clusters within what is termed the barrel hollows, with the number of TCA fibers between these domains being reduced. Therefore, during thalamocortical circuit formation, TCAs have the opportunity to make contacts with neurons of different cortical layers spanning a large region. To reveal electroporated cells in the brain the NT-probe was co-expressed with nuc-mCherry and the CT-probe was co-expressed with nuc-Azurite (Fig. 6D). In an electroporated P8 mouse brain, reconstituted GFP was observed in the cortex in a scattered manner (Fig. 6C, E). All GFP-positive cortical neurons showed the nuc-mCherry signal, however, none of them displayed Azurite-positive nuclei (Fig. 6E and data not shown). This suggests that the signals in these cortical NT neurons were specifically from GFP reconstituted by transient contact between TCAs and cortical neurons during development. Importantly, in contrast with the *d*GRAPHIC signal in the mature brain, which was restricted to layer IV (Fig. 4 and data not shown), labeling the thalamocortical circuit in the immature brain led to GFP-positive neurons in multiple cortical layers. This suggests that GFP reconstitution occurred during transient contacts between the TCAs and neurons in the other cortical layers prior to final axonal organization. These results suggest that *d*GRAPHIC is applicable to labeling of both mature and developing neuronal circuits to reveal their transient contacts.

## Discussion

Here, we have generated a novel BiFC-based fluorescent probe system, *d*GRAPHIC, that allows the labeling of cell morphology based on contact-dependent GFP reconstitution. By removing the leucine zipper domain from GRAPHIC, unilateral diffusion of reconstituted GFP was observed, allowing the preferential labeling of contacted cells in one direction. We demonstrated that *d*GRAPHIC could be applied in many different kinds of biological contexts, such as in epithelial cell culture, *Xenopus* blastomeres, dissociated hippocampal culture, and adult and young neural circuits, with relatively simple transfection methods.

All other BiFC-based probes for detecting neuronal connectivity, such as GRASP (including mGRASP), involve a pair of synaptic membrane proteins and clearly indicate precise synaptic sites ^13,14^. However, given the diversity and complexity of both connectivity and cell types, the ability to label the entire morphology of connected neurons is a great advantage when dissecting specific neuronal circuits. We have demonstrated the ability of *d*GRAPHIC to reveal the morphology of axons, dendrites, and cell bodies of connected neurons. Molecular and viral tracers also have this ability, however, in addition to simpler genetic approaches, *d*GRAPHIC has additional advantages. The general molecular structure of GRAPHIC does not contain specific domains for pre- or postsynaptic targeting, therefore, NT and CT probes can be expressed in both presynaptic neurons and postsynaptic neurons, which makes anterograde or retrograde labeling possible by exchanging probe expression. Further, *d*GRAPHIC has no specific domain for cell-specific expression, which allows us to equally label neuronal connections among different neuronal cell types and detect not only neuron-neuron interactions, but also neuron-glia or glia-glia interactions. *d*GRAPHIC has a relatively simple design, containing only a GPI anchor, GFP fragment, and small artificial domains in extracellular region. Moreover, in our previous study, we tested whether GRAPHIC artificially strengthens cell adhesion ^17^. We estimated cell detachment rates of LLCPK1 cells with and without expression of GRAPHIC molecules, and could not observe a significant effect of GRAPHIC on cell adhesion. Considering that formation of juxta-membrane complexes and anchoring to the cytoskeleton are necessary for adhesion molecules, such as cadherins, GRAPHIC is unlikely to generate significant force at cell–cell contact sites due to its lack of intracellular domain. Because *d*GRAPHIC contains the same backbone as GRAPHIC, it is very likely that *d*GRAPHIC does not send any intercellular signals. At least, we did not notice any extra cell death or abnormal brain development by overexpression of *d*GRAPHIC in vivo.

Signal diffusion on the contacted cell membrane indicates another possibility for *d*GRAPHIC application. In our previous work, we showed that GRAPHIC has specific GFP reconstitution activity at cell–cell contact sites, and its signal complex is stable and can remain on either contacted cell surface until GFP is degraded, even after dissolution of intercellular contacts ^17^. This suggests that *d*GRAPHIC can detect past transient contact even if the connection is not maintained. This “memory dye” characteristic will be useful for investigating synapse pruning and axon refinement, such as the development of the retinal superior colliculus circuit ^24^, and trace the footprints of migratory cells, such as interneuron migration in the developing telencephalon ^25^.

In our previous paper, we demonstrated that GRAPHIC color variants can be generated ^17^. In the same way, it is possible to generate color variants with *d*GRAPHIC, which will make it possible to reveal multiple convergent inputs and their neuron selectivity. *d*GRAPHIC enables us to design tailor-made assay systems for detecting many types of intercellular connectivity, highlighted here for the nervous system. Moreover, *d*GRAPHIC was designed not only for the nervous system, because theoretically it can be applied to detection of any intercellular contact. For example, using its “memory dye” character, immune-educated T-cells may be visualized in primary or secondary lymphoid organs, and additionally in distal blood vessels or sites of activation. Further, because breakdown of cell–cell contacts is one of the hallmarks of carcinogenesis, *d*GRAPHIC may be a simple and easy method for screening carcinogens. Therefore, together with GRAPHIC, *d*GRAPHIC will be a powerful tool to reveal intercellular contacts that are essential for precise organ morphogenesis, function, and maintenance.

## Methods

### Animals

All procedures were approved by the RIKEN Institutional DNA [2018-010(7)] and Animal Care and Use Committee [W2020-2-021(2)] and complied with all relevant ethical regulations.

### Probe cDNA constructs

GRAPHIC was generated as described in a previous paper^17^. LZA or LZB, which was inserted between the sfGFP fragment and membrane-associated domains, was removed to generate *d*GRAPHIC. Spacer domains (cDNA sequence: 5′-GGTGGAGGCGGTTCAGGCGGAGGTGGCTCTGGCGGTGGCGGATCG-3′) were inserted between the sfGFP fragment and membrane-associated domain. To identify transfected neurons, probe molecules were fused to mCherry, H2B-mCherry, or H2B-Azurite protein with self-cleavable T2A peptide (DNA sequence: 5′-GAGGGCAGAGGAAGTCTTCTAACATGCGGTGACGTCGAGGAGAATCCTGGCCCA-3′).

### Cell cultures

LLCPK1 cells were cultured in DMEM (low glucose, Wako) containing 10% fetal bovine serum (FBS, Serum Source International). H2B-mCherry- or H2B-Azurite-expressing monoclonal LLCPK1 cells were first generated using the lentivirus system. Probe-expressing, nuclei-labeled LLCPK1 cells were generated with lentivirus infections. HEK293T cells (RIKEN Cell Bank) were cultured in DMEM (high glucose, Wako) medium containing 10% FBS. All cell lines were cultured at 37 °C under 5% CO_2._

### *Xenopus* blastomere injection

Injection of mRNA into *Xenopus* 8-cell stage blastomeres is performed as described previously ^18^. The mRNA of the NT probe is injected into the right animal-smaller blastomere, and the mRNA of GFP 8–11 and H2B-Cherry is injected into the left animal-smaller blastomere. Embryos are incubated until they reach the early neurula stage and fluorescence is observed.

### Hippocampus dissociated culture

Dissociated hippocampal neurons were prepared from Sprague Dawley rat embryos (E18-19). Dissected hippocampus was digested with 0.3% papain (Wako), 1 mM EDTA, and 100 U/ml DNaseI (Roche), then plated on poly-L-lysine (peptide)-coated glass-bottomed dishes (Iwaki) in culture medium A. Culture medium A is MEM (Sigma) containing 20% FBS and N2 supplement (Life Technologies). On the next day, the culture medium was changed to culture medium B (MEM containing 2% FBS and N2 supplement), then half of the spent culture medium was replaced with fresh culture medium B every 3 days.

### Electroporation of dissociated hippocampal neurons

Just after dissociation of hippocampal neurons, electroporation of the dissociated neurons was carried out with the Nucleofector 2b system (Rat Neuron Nucleofector Kit, program O-003; Lonza). For stable expression over several weeks, we used a genome integration system employing Tol2 transposase. A cDNA encoding probe molecule and fluorescent label were cloned into a pT2K-CAGGS vector, and the plasmid was co-electroporated with pCAGGS-T2TP.

### In utero electroporation

In utero electroporation (IUE) of ICR mice were carried out as described previously. For double IUE, we used pCAGGS vectors encoding probe molecules and fluorescent nuclear labels. For visualization of adult mouse neuronal connection, we used a genome integration system employing Tol2 transposase, as mentioned in **Electroporation of dissociated hippocampal neurons**. All IUE was done in the right side of embryonic mouse brains.

### Adeno-associated virus injection

For AAV production, we used AAV-DJ/8 Helper Free Expression System (Cell Biolabs). DJ/8 is an artificially generated AAV serotype. A cDNA encoding probe molecule and fluorescent label (GPI-sfGFP 8-11∆C10 T2A mCherry) were cloned into a pAAV-MCS vector. To produce AAV solution, pAAV-MCS was co-transfected with pAAV-DJ, which supplies AAV-2 Rep and AAV-DJ Cap, and pHelper, which provides most of the adenovirus gene products required for the AAV production into the 293AAV Cell Line (Cell Biolabs) by 293fectin (Life Technologies). The AAV solution was concentrated by ultracentrifugation (87,000 *g*, 4 °C, 2 h) with 30% sucrose barrier.

The AAV was injected into the right hemisphere thalamus and cortex by stereotaxic injection. Injection of volume of AAV solutions was 500 nl per location, and the rate was 200 nl/min. After the injection, the needle remained in place for an additional 2.5 min before removal.

### Imaging of cultured cells

For time lapse imaging, cells were grown on a glass-bottomed dish in DMEMgfp (Evrogen) containing 10% FBS and 1 × GlutaMAX (Life Technologies). For timepoints, culture mediums of confluent LLCPK1 cells were replaced with Hank’s Balanced Salt Solution without phenol red (Nacalai) just before imaging. Most cultured cell images were collected with inverted Olympus IX-81 or -83 microscopes and Hamamatsu ORCA-Flash 4.0 camera. For investigation of subcellular distribution of NT-probe molecules or reconstituted signals, immunostained or fixed cells were subjected to confocal imaging with an FV1000 Olympus microscope. Dissociated hippocampal neurons were also fixed with 4% paraformaldehyde in PBS, and replaced with PBS before being subjected to confocal imaging with the FV1000 microscope. Images were processed in ImageJ and Adobe Photoshop.

### Imaging of brain tissues

All electroporated and AAV-injected ICR mice were anesthetized with a lethal dose of pentobarbitone (100 mg/kg), and after three failed attempts to elicit a foot withdrawal reflex, the animals were transcardially perfused with 4% paraformaldehyde in PBS. Brains were collected and sectioned in the coronal plane with a Leica VT1000S vibratome at 100–120 µm. Confocal images were collected with the FV1000 microscope. Low-magnification images in Fig. 4 were collected with an Olympus IX83 microscope and Hamamatsu ORCA-Flash 4.0 camera. Images were processed in ImageJ and Adobe Photoshop.

## Supplementary information

Supplementary file 1.

Supplementary file 2.

Supplementary file 3.
