# Supplementary material for: Diffusible GRAPHIC to visualize morphology of cells after specific cell–cell contact
Source: Sci Rep. 2020 Sep 2;10:14437. doi: 10.1038/s41598-020-71474-0 (PMC7468259; doi:10.1038/s41598-020-71474-0)
Supplement: Supplementary file 1 [file 41598_2020_71474_MOESM1_ESM.docx]

Supplementary figure 1

A red nucleus (labeled by H2B-mCherry) indicates an NT cell, and a blue nucleus (labeled by H2B-Azurite) indicates a CT cell. (A) NT probe and CT-probe connected to PDGFR transmembrane domain (PDGFR-TM). (B) Reconstituted GFP was observed only in the CT cells. (C) Combination of NT probe with PDGFR-TM and CT-probe with GPI. (D) Reconstituted GFP was observed only in the CT cells. Scale bar in B, 40µm.

Supplementary figure 2

AAVs encoding NT probes and CT probes (co-expression with cytosolic mCherry) were stereotaxically injected into the S1 cortex and VB, respectively as like Fig 4. After several weeks, the injected brains were sectioned and observed. In coronal sections, Nissl staining (A) higher magnification (B) the GFP signal was observed in the cortex. Position of Layer IV is indicated by white dotted line. Scale bar, 200µm for A and 100µm for B and C.

Supplemental figure 3

*In utero* electroporation was performed at E13.5 to express NT probes (red nuclear label) in cortical layer IV. After the electroporated mice had grown to adult age (about 2 months old), AAV encoding the CT probe (cytosolic mCherry) was stereotaxically injected into the VB. Reconstituted GFP and nucleic mCherry signals were observed in layer IV, DAPI staining in blue. Scale bar, 100 μm.

**Supplementary movie 1**

Dynamics of *d*GRAPHIC signals during the establishment of intercellular contact of epithelial cells. This movie shows the signal distribution of *d*GRAPHIC and the change in its intensity during establishment of the intercellular contact between n-GRAPHIC-expressing LLCPK1 cells (red nuclei) and c-GRAPHIC-expressing LLCPK1 cells (blue nuclei). Length of movie, 35 h.
