# Supplementary material for: Diffusible GRAPHIC to visualize morphology of cells after specific cell–cell contact
Source: Sci Rep. 2020 Sep 2;10:14437. doi: 10.1038/s41598-020-71474-0 (PMC7468259; doi:10.1038/s41598-020-71474-0)
Supplement: Supplementary file 2 [file 41598_2020_71474_MOESM2_ESM.pdf]

Diffusible GRAPHIC to visualize morphology of cells after specific cell-cell contact

Nagatoki Kinoshita, Arthur JY Huang, Thomas J McHugh, Atsushi Miyawaki and Tomomi Shimogori

Supplementary Figures

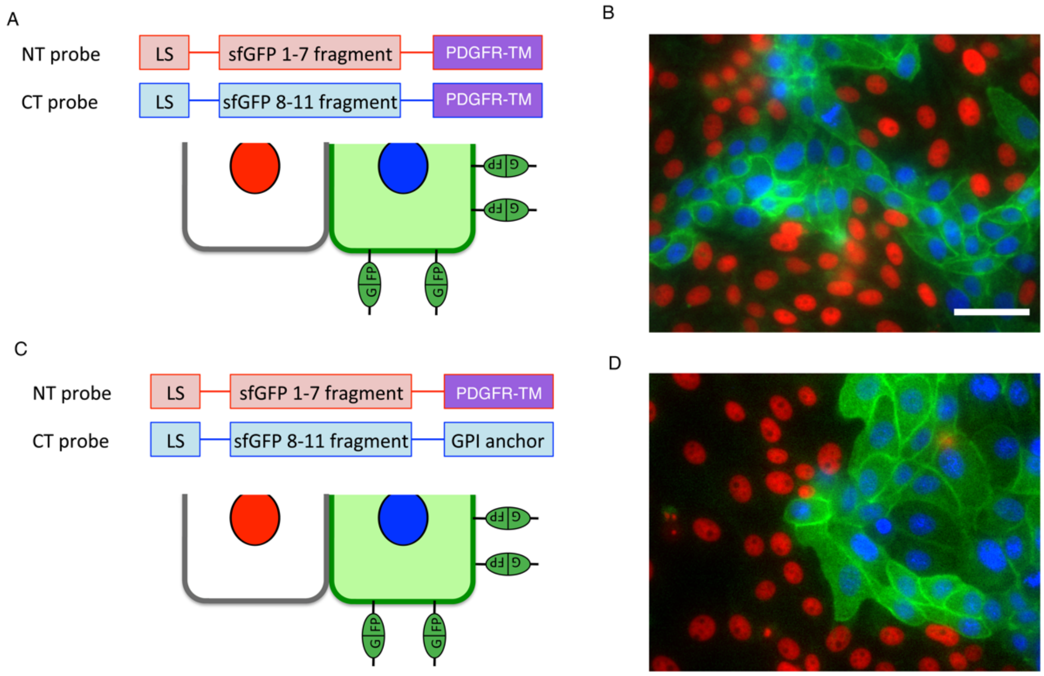

sFig 1 Kinoshita et al.,

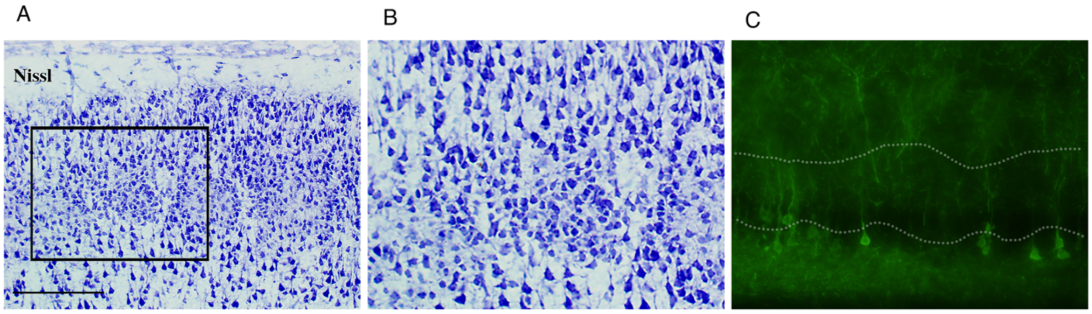

sFig 2

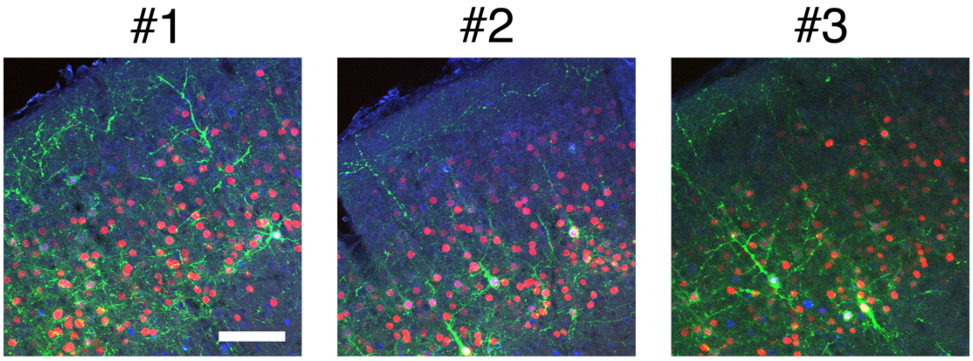

sFig 3 Kinoshita et al.,
